# Suicide warning signs that are challenging to recognize: a psychological autopsy study of Korean adolescents

**DOI:** 10.1186/s13034-024-00731-1

**Published:** 2024-03-25

**Authors:** Yeon Jung Lee, Yong-Sil Kweon, Yun Hyong Kang, Kyung Hee Yoon, Mi-Sun Lee, Soo-Young Bhang, Hyun Ju Hong

**Affiliations:** 1 Department of Psychiatry, College of Medicine, Soonchunhyang University Seoul Hospital, Seoul, Republic of Korea; 2 Department of Psychiatry, College of Medicine, The Catholic University, Seoul, Republic of Korea; 3 Doctors Seoul Mental Clinic, Seoul, Republic of Korea; 4 School Mental Health Resources and Research Center, Seoul, Republic of Korea; 5 Department of Preventive Medicine, College of Medicine, The Catholic University, Seoul, Republic of Korea; 6 Department of Psychiatry, Nowon Eulji University Hospital, Eulji University School of Medicine, Seoul, Republic of Korea; 7 Eulji Psychiatry and Medical Science Center, Eulji University, Seongnam-si, Gyeonggi-do Republic of Korea; 8 Suicide and School Mental Health Institute, Anyang, Republic of Korea; 9 Department of Psychiatry, Hallym University Sacred Heart Hospital, 22 Gwanpyeong-ro 170 beon-gil, Dongan-gu, Anyang, 14068 Republic of Korea

**Keywords:** Suicide, Adolescent, Autopsy

## Abstract

**Background:**

In South Korea, the leading cause of youth death has been suicide for about 20 years. In this study, we conducted a multi-method psychological autopsy to identify the psychiatric diagnosis, developmental history, personality traits, family history, school life, warning signs of suicide, and risk factors related to suicide for the first time in Korea.

**Methods:**

This was a postmortem, retrospective, and descriptive study of 36 adolescents who died by suicide between August 2015 and July 2021 in South Korea. We obtained qualitative and quantitative data from the Korean Psychological Autopsy of Adolescent, conducted by the Suicide and School Mental Health Institute, the official student mental health policy-focused research institute of the Korean Ministry of Education.

**Results:**

The adolescents comprised equal percentage of girls and boys. Approximately half of the deaths (55.6%) occurred at home and most (72.2%) involved jumping from a height. Most of the adolescents (97.2%) had one major psychiatric disorder before death, with depressive disorder being the most prevalent (75%). They were at a high risk for internet addiction before death. The most common personality trait was avoidance (28.6%), followed by submissiveness (27.3%). Half of the parents reported that the adolescents were satisfied with their school life and the teachers observed that they had no behavioral problems. One year before death, seven (19.4%) adolescents injured themselves and five (13.9%) had attempted suicide. Most of the deceased (80.6%) had expressed suicide warning signs to their families within one year before death. Adolescents had a long experience of family-related adverse events.

**Conclusions:**

Most of the adolescents had mental health disorders and expressed suicidal intentions using verbal and nonverbal signs. However, it was challenging for families to recognize the risk signs because of adolescents’ personality traits or a good school life. To prevent adolescent suicide, adolescents, parents, and teachers need to be educated to recognize signs of suicide warning signs and equipped to guide adolescents to appropriate care.

**Supplementary Information:**

The online version contains supplementary material available at 10.1186/s13034-024-00731-1.

## Background

Adolescent suicide is a serious public health issue worldwide. Globally, 703,000 people die by suicide each year, and more than one in every 100 deaths in 2019 resulted from suicide [1]. Suicide is the fourth leading cause of death among individuals aged 15 to 29 years [1]. In South Korea, suicide has been the leading cause of death, with 6.5 deaths per 100,000 people among adolescents aged 10 to 19 in 2009. The leading cause of youth death from 2009 to 2022 was suicide, except in 2014 when the Sewol Ferry disaster occurred [2–4]. The Korean Annual Nationwide Survey initiated a pilot project from 2007 to 2011 to screen students’ mental health and has implemented suicide prevention program nationwide since 2012 [5]. As a result, the adolescent suicide rate dropped to 4.7 per 100,000 in 2016, increased to 5.8 per 100,000 in 2017, and further rose to 7.2 deaths per 100,000 in 2022 [2, 3].

Suicide is caused by the complex interrelationships between gender, familial genetic factors, previous suicidal behaviors, history of mental illness and physical disorders, personality, peer relationships, family relationships, school environment, and stressful life events [6–11]. The American Association of Suicidology presented risk factors based on the lives of people who died by suicide, for suicide risk assessment and formulation purposes [8, 12, 13]. Chronic risk factors included male sex, old age, mental disorders, history of trauma or abuse, low self-esteem, lack of self or familial acceptance of sexual orientation, and perfectionism. Acute risk factors included suicide ideation, current self-harm behavior, recent suicide attempts, anhedonia, hopelessness, and excessive risk-taking behaviors. Triggering stimuli included any actual or anticipated event that caused or threatened shame, guilt, or despair, and recent exposure to another’s suicide. Nonetheless, due to the difficulty in predicting suicide attempts or deaths resulting from distal risk factors, researchers have tried to focus on more proximal risk factors [14, 15].

A psychological autopsy can be an effective way to study the complex dynamics causing suicide [16]. “Psychological autopsy” is a technique consisting of epidemiological and case study methods and is the most valuable and informative procedure for reconstructing a death’s social and psychological characteristics by interviewing those close to the deceased person [17, 18]. Although the technique has limitations of being retrospective and involving a risk of recall bias, it is the most efficient way to determine the cause of suicide deaths [19, 20]. The method may be more useful to study adolescent suicides than adult suicides because there are more opportunities to interview their entire family, friends, teachers, and close acquaintances [19, 20].

Less adolescents than adults die by suicide, and it is challenging to conduct a psychological autopsy with the consent of a bereaved family. Therefore, suicide of adolescents have not been extensively studied using psychological autopsies. Previous studies [6, 10, 21–23] related to adolescent suicide have found characteristic differences depending on country and culture. In Western countries, adolescent suicide is more common in boys [21, 22, 24, 25], and is related to impulsivity [10, 26, 27], and excessive drinking or illegal drug abuse history [21, 28]. In contrast, in Eastern countries, substance abuse rates are lower [29], and gender differences are lesser [6, 10, 22, 25]. However, the suicide method is different in each country due to accessibility, with firearms being the most common method in the United States [30] and falling from a height, in areas with high buildings [29, 31]. Therefore, to tailor suicide prevention programs to South Korean adolescents, it is necessary to identify the characteristics unique to Korean adolescents who died by suicide, along with variables previously identified as risk factors [6, 10].

To the best of our knowledge, no psychological autopsy studies have been conducted on Korean adolescents who have died by suicide. This is the first preliminary study that used psychological autopsy data of Korean adolescents. The main objectives were: (1) to investigate psychiatric diagnosis, developmental history, personality traits, family history, school life, and warning signs of suicide, as well as Internet addiction; (2) to examine the risk and protective factors for suicide; and (3) to analyze the gender differences in adolescent suicide.

## Methods

### Study design and procedures

Psychological autopsies of 36 adolescents who died by suicide were performed by involving their 36 parents (5 fathers and 31 mothers). A multi-method approach included qualitative and quantitative data collection through semi-structured interviews using the Korean Psychological Autopsy Checklist for Adolescents (K-PAC-A) [32] and standardized psychological questionnaires. The interviews were conducted at the participants’ homes or in nearby conference rooms that were quiet and well-insulated.

The research team comprised psychiatrists, psychologists, and researchers in the mental health field. More than 90% of the primary interviewers were licensed child and adolescent psychiatrists. Primary interviewers, trained psychiatrists, and assistant interviewers with a master’s degree or doctorate conducted each interview for three hours. The interviewers did not have any prior information about the adolescents and interviewees. The assistant interviewer explained the purpose and method of standardized psychological questionnaires to the participants. Quantitative data from questionnaires and quantitative and qualitative data from interviews were collected. One primary interviewer interviewed one parent in 28 cases, and two primary interviewers interviewed two parents each in eight cases. When both parents participated in the psychological autopsy, data from one parent who had more accurate and objective information about their offspring were selected through a research team meeting. Follow-up calls were made two weeks later to assess changes in the participants’ emotions, physical conditions, and activities, and to provide necessary support.

The interviews were recorded with the participants’ consent. Assistant interviewers wrote the content verbatim and created a database within a month. The primary and assistant interviewers drafted the “In-depth analysis form of the adolescent psychological autopsy” (Appendix 2). Through research team meetings (Appendix 3), the final diagnosis, risk, and protective factors were confirmed in addition to the psychodynamic formulation leading to suicide. The psychodynamic formulation in the study involved exploring in the context of their life how an individual interacts with their environment, develops suicidal thoughts, attempts suicide, and ultimately dies by suicide [33].

### Participants

This study used the data of 36 Korean adolescents who had died by suicide collected from August 2015 to July 2021 by the Suicide and School Mental Health Institute, the official student mental health policy-focused research institute of the Korean Ministry of Education that aims to build and examine national data on adolescent suicide. The institute advertised for the psychological autopsy of adolescents to mental health institutes in the country, including the Korean National Police Agency, the Korea Foundation for Suicide Prevention, local mental health welfare centers, the Korean Suicide Prevention Centers, the Metropolitan and Provincial Offices of Education, and mental health hospitals and clinics for children and adolescents, to recruit the participants. The inclusion criteria were adolescents aged 10–19 years who died by suicide, both parents agreed for a psychological autopsy, and at least one month of grief had passed [34–36]. Cases with legal issues related to suicide or disagreement with both parents regarding the psychological autopsy were excluded. If one parent could not attend a psychological autopsy because of divorce, separation, or death, we required an agreement from the single parent who could attend. The present study was approved by the Institutional Review Board of Hallym University Sacred Heart Hospital (2016 − 1044). Informed consent was submitted by all parents when they were enrolled.

### Measures

#### Korean psychological autopsy checklist for adolescents (K-PAC-A)

The Korean Psychological Autopsy Checklist for Adolescents (K-PAC-A) is a psychological autopsy tool that evaluates situations before suicide and suicide-related factors in adolescents who have died by suicide [32]. It uses a semi-structured clinical interview with experts and a questionnaire administered to the parents. The K-PAC-A comprises three parts (Appendix 1): (I) information about the interviewee, who is a suicide survivor, such as sociodemographic information and psychological and adaptive status after the adolescent’s death; (II) information about the adolescent, such as sociodemographic information, age at the time of death, method and place of suicide, suicide warning signs, history of suicidal behaviors, developmental history, adverse events based on the developmental stage, personality traits, relationship with parents, peers, and teachers, family-related information, school life related information, and physical and mental health; and (III) psychological assessment of the adolescent and the parents, using diagnostic interviews or standardized questionnaires. According to the Korean Psychological Autopsy Center, suicide warning signs, that is, signals indicating a high possibility of suicide, include linguistic, behavioral, and situational signs [37, 38]. In this study, factors known as “suicide warning signs” in adolescents were categorized into verbal, behavioral, and/or emotional signs (Appendix 4) [32]. The primary interviewer asked the interviewees whether the adolescents displayed verbal, behavioral, and/or emotional signs that indicated their suicidal crisis before death, and explained warning signs using examples (Appendix 4) [37, 38]. Information on signs identified through the interviews was collected and placed in the suicide warning signs categories. We defined protective factors as factors that act as a buffer for adolescents under challenging situations corresponding to risk factors before death. Protective factors were identified according to the psychological autopsy information provided by the interviewees. Risk and protective factors were summarized using words as objectively as possible, focusing on situations or events. A word cloud provided a visual representation of risk and protective factors organized as words.

#### Kiddie-schedule for affective disorders and Schizophrenia-Present and Lifetime-Korean Version (K-SADS-PL-K)

The Kiddie-Schedule for Affective Disorders and Schizophrenia-Present and Lifetime (K-SADS-PL) evaluates lifetime and current episodes of children and adolescents based on the Diagnostic and Statistical Manual of Mental Disorders-4th edition (DSM-IV) through semi-structured interviews [39]. Kim et al. standardized the Korean version of the K-SADS-PL [40]. It involves interviewing parents and children to gather information. The final diagnosis combines parents’ reports of observable behaviors and children’s reports of subjective experiences. However, our diagnoses were based on only parents’ reports of observable behaviors and estimated emotions three months before the adolescent’s death. Psychiatric diagnosis at the time of death is based on K-SADS-PL. However, since only parent interviews were possible, a final diagnosis based on DSM-5 was made through a research meeting based on various information obtained from psychological autopsy interviews. The primary diagnosis is defined as the one causing the most functional impairment, and the others are classified as comorbidities at the time of death.

#### Korean version of the Barratt Impulsiveness Scale-11 (K-BIS-11)

The Korean version of the Barratt Impulsiveness Scale-11 **(**BIS-11) is a self-report questionnaire comprising 30 items revised using the Barratt Impulsiveness Scale [41]. In this study, parent survivors of suicide completed the questionnaire to evaluate the severity of the deceased’s impulsivity, a suicide risk factor [42]. It was explained that the evaluation would be based on the judgment of the bereaved parents. Each item was rated on a 4-point Likert scale, with total scores ranging from 30 to 120. The higher the total score, the higher the impulsiveness. Although this is a self-report questionnaire, the parents completed it; therefore, caution is required when interpreting the results. Heo et al. [43] standardized the Korean version of the BIS-11. Cronbach’s alpha was 0.80, and the test-retest reliability was 0.95 [43].

#### Korean version of Beck Depression Inventory-II (K-BDI-II)

The Beck Depression Inventory-II (BDI-II) is a 21-item self-report questionnaire that assesses the presence and severity of depressive symptoms (cognitive, emotional, motivational, and physical) over past two weeks [44]. This questionnaire was intended to investigate why bereaved parents often do not recognize symptoms of depression, and it was evaluated based on their judgment. In this study, parent survivors of suicide completed the questionnaire to evaluate the severity of the deceased’s depression two weeks before death. Items are rated on a 4-point Likert scale, and the total score is evaluated as 10–15 = mild, 16–23 = moderate, and 24–63 = severe depression. Although this is a self-report questionnaire, it was completed by the parents; therefore, caution is required when interpreting the results. Sung et al. [45] standardized the Korean version of BDI-II (K-BDI-II), and obtained a Cronbach’s alpha of > 0.80. In this study, parents responded to the scale on the severity of depression in the deceased.

#### Korean attention Deficit/Hyperactivity disorder (ADHD) rating scale-IV (K-ARS-IV)

The attention deficit/hyperactivity disorder (ADHD) rating scale-IV (ARS-IV) is used by parents or teachers to assess hyperactivity and problematic behaviors in children and adolescents [46]. It comprises 18 items according to the ADHD diagnostic criteria of the DSM-IV. Each item is rated on a 4-point Likert scale. Parents completed it to evaluate the hyperactivity and problematic behaviors of the deceased. A total score of 19 or higher was defined as clinically-significant ADHD symptoms. Jang et al. standardized the Korean version of the ARS-IV, in which Cronbach’s alpha was 0.74 − 1 and validity was 0.06 − 0.59 [47].

#### Internet addiction proneness scale for Youth: Observer Rating Scale (KO scale)

The Internet Addiction Proneness Scale for Youth: Observer Rating Scale **(**KO scale) evaluates Internet addiction in adolescents based on teachers’ and parents’ observations [48] to compensate for the shortcomings of the Internet Addiction Proneness Scale (K-scale)—a self-questionnaire [49, 50]. The KO scale evaluates the main symptoms of Internet addiction, including compulsive use and obsession, tolerance and withdrawal, behavioral symptoms, daily life dysfunction, deviant behavior, and behavioral symptoms of hypothetical interpersonal orientation. A total score of > 10 implies a high risk for Internet addiction. Lee et al. standardized the KO scale, and the Cronbach’s alpha was 0.88–0.89 [48].

#### Adjective scale to assess the personality types

The Adjective Scale to Assess Personality Types [51] was developed using Korean adjectives based on Millon’s personality and personality disorder model [52]. This scale evaluates nine personality dimensions (asocial, avoidant, submissive, gregarious, narcissistic, aggressive, conforming, negativistic, and problem indicators) according to different behavioral patterns, based on the theory that natural languages help describe individual differences in human tendencies. The scale comprises 178 items that describe personality types and can be evaluated by the individual and observers to explain subjective personality types. Each item is answered as “Yes” or “No.” Problem indicators include the schizotypal, borderline, and paranoid personality types. Personality types were confirmed by the ratio of the number of “Yes” to the total number of questions for each dimension. Choi et al. standardized the scale; the Cronbach’s alpha was 0.71 − 0.87 [51].

### Statistical analysis

This cross-sectional descriptive study evaluated two sections related to adolescents and their family members. For adolescents, we evaluated (1) demographic, psychological, and clinical characteristics at the time of death; (2) suicidal warning signs before death; (3) word clouds of risk and protective factors; (4) information about school life; and (5) adverse events based on the developmental stage. The risk and protective factors for the adolescents were visualized as word clouds based on the psychodynamic formulation organized through a research team meeting. The risk factors were subdivided according to the timeline before death. Of them, those occurring within approximately seven days before the date of death were defined as “immediate triggers”; those occurring from a week to within two months as “acute risk factors”; and those lasting from two months to two years as “chronic risk factors.” Protective factors were those that could reduce the impact of a risk factor or the likelihood of adverse outcomeslasting from two months before death to two years. For their parents and family members, family mental health history was collected from the parents. To investigate gender differences, the Mann-Whitney U test and Fisher’s exact test were used. All data were analyzed using IBM SPSS Statistics Version 28.0 (IBM Corp., Armonk, NY, USA). A *p*-value < 0.05 was considered statistically significant.

## Results

### Demographic and psychological characteristics of the adolescents

The mean age of the adolescents was 16.1 years (SD 2.0; Table 1). For most of them, home was the place of death. The most common method of suicide was jumping from a height (72.2%), followed by hanging (16.7%), poisoning with gas or vapor (8.3%), and drowning (2.78; Table 1). The majority did not ask anyone for help before death (97.2%).

**Table 1:** Demographic characteristics at the time of death in the adolescents

Variables		Total (36, 100%)	Male (18, 50%)	Female (18, 50%)	p value
		M ± SD / N (%)			
Age		16.1 ± 2.0	16 ± 1.9	16.3 ± 2.0	0.519
School enrollment					> 0.99
Yes 33 (91.7%)	Elementary school	2 (5.6%)	1 (2.8%)	1 (2.8%)	
	Middle school	7 (19.4%)	3 (8.3%)	4 (11.1%)	
	High school	23 (63.9%)	12 (33.3%)	11 (30.6%)	
	Alternative high school	1 (2.8%)	0 (0%)	1 (2.8%)	
No 3 (8.3%)	Middle school graduation	1 (2.8%)	1 (2.8%)	0 (0%)	
	High school graduation	2 (5.6%)	1 (2.8%)	1 (2.8%)	
Family form					0.62
Living with two parents		24 (66.7%)	13(36.1%)	11 (30.6%)	
Living with a parent 12 (33.3%)	Due to parent’s divorce	10 (27.8%)	5 (13.9%)	5 (13.9%)	
	Due to father’s death	2 (5.6%)	0 (0%)	2 (5.6%)	
Place of suicide					0.07
Home		20 (55.6%)	9 (25%)	11 (3 0.6%)	
Public place		9 (25%)	5 (13.9%)	4 (11.1%)	
School		2 (5.6%)	2 (5.6%)	0 (0%)	
Hospital		1(2.8%)	0 (0%)	1 (2.8%)	
Others		4 (11.1%)	2 (5.6%)	2 (5.6%)	
Methods of suicide					0.258
Jumping from height		26 (72.2%)	12 (33.3%)	14 (38.9%)	
Hanging or strangulation		6 (16.7%)	3 (8.3%)	3 (8.3%)	
Poisoning by gas or vapor		3 (8.3%)	3 (8.3%)	0 (0%)	
Drowning		1 (2.8%)	0 (0%)	1 (2.8%)	
Drinking					> 0.99
Yes		2 (5.6%)	1 (2.8%)	1 (2.8%)	
No		33 (91.7%)	17 (47.2%)	16 (44.4%)	
Unknown		1 (2.8%)	0	1 (2.8%)	
Asking for help after suicidal behavior before death					0.691
Yes		0 (0%)	0 (0%)	0 (0%)	
No		35 (97.2%)	17 (47.2%)	18 (50%)	
Unknown		1 (2.8%)	1 (2.8%)	0 (0%)	

M: mean; SD: standard deviation; N: number.

As shown in Table 2, most adolescents (97.2%) were diagnosed with a major mental disorder in the three months before death and a significant proportion (80.6%), with affective disorder. More than half of them (55.6%) were diagnosed with comorbidities. The most common comorbidity was depressive disorder, followed by generalized anxiety disorder.

**Table 2:** Psychological characteristics at the time of death in adolescents reported by parents (*N* = 36)

Variables				Total (36, 100%)	Male (18, 50%)	Female (18, 50%)	p value
				M ± SD / N (%)			
Diagnosis (DSM-5)							0.905
Primary diagnosis (35, 97.2%)	Affective disorder 29 (80.6%)	Depressive disorders 27 (75%)	Major depressive disorder	16 (44.4%)	7 (19.4%)	9 (25%)	
			Unspecified depressive disorder	7 (19.4%)	3 (8.3%)	4 (11.1%)	
			Persistent depressive disorder	4 (11.1%)	3 (8.3%)	1 (2.8%)	
		Bipolar II disorder		2 (5.6%)	1 (2.8%)	1 (2.8%)	
		Schizophrenia		2 (5.6%)	1 (2.8%)	1 (2.8%)	
		Attention deficit/hyperactivity disorder		1 (2.8%)	1 (2.8%)	1 (2.8%)	
		Acute stress disorder		1 (2.8%)	1 (2.8%)	1 (2.8%)	
		Other specified anxiety disorder		1 (2.8%)	1 (2.8%)	0 (0%)	
		Intellectual disabilities		1 (2.8%)	0 (0%)	1 (2.8%)	
No major mental disorder				1 (2.8%)	0 (0%)	1 (2.8%)	
							0.94
Comorbidities (20, 55.6%)	Depressive disorder (4, 11.1%)		Major depressive disorder	1 (2.8%)	0 (0%)	1 (2.8%)	
			Persistent depressive disorder	2 (5.6%)	0 (0%)	2 (5.6%)	
			Unspecified depressive disorder	1 (2.8%)	1 (2.8%)	1 (2.8%)	
	Generalized anxiety disorder			3 (8.3%)	1 (2.8%)	2 (5.6%)	
	Attention Deficit/Hyperactivity Disorder			2 (5.6%)	1 (2.8%)	1 (2.8%)	
	Oppositional defiant disorder			2 (5.6%)	1 (2.8%)	1 (2.8%)	
	Adjustment disorder			2 (5.6%)	1 (2.8%)	1 (2.8%)	
	Obsessive compulsive disorder			1 (2.8%)	1 (2.8%)	0 (0%)	
	Internet gaming disorder			1 (2.8%)	1 (2.8%)	0 (0%)	
	Alcohol abuse			1 (2.8%)	0 (0%)	1 (2.8%)	
	Schizophrenia			1 (2.8%)	0 (0%)	1 (2.8%)	
	Social phobia			1 (2.8%)	1 (2.8%)	0 (0%)	
	Mental retardation			1 (2.8%)	1 (2.8%)	0 (0%)	

M: mean; SD: standard deviation; N: number; DSM-5: Diagnostic and Statistical Manual of Mental Disorders-5th edition.

The mean depression and Internet addiction scores of the adolescents were 24.19 (SD 13.63) and 31.73 (SD 6.05), respectively, indicating severe depression and a high risk for Internet addiction (Table 3). Regarding personality traits, female adolescents exhibited significantly higher levels of avoidant and submissive traits, negativity, and problem indicators than male adolescents. The rate of self-injury among female adolescents before dying by suicide was significantly higher than that among male adolescents.

Psychiatric intervention was experienced by 47.2%. Three adolescents (8.3%) received inpatient treatments at the Department of Psychiatry and only three (8.3%) received regular medication treatments.

**Table 3:** Clinical characteristics at the time of death in adolescents reported by parents (*N* = 36)

Variables		Total (36, 100%)	Male (18, 50%)	Female (18, 50%)	p value
		M ± SD / N (%)			
Psychological assessment					
K-BDI-II		24.2 ± 13.6	22.1 ± 14.9	26.1 ± 12.6	0.331
K-ARS		10.8 ± 10.3	9.9 ± 8.3	11.6 ± 12.0	0.794
K-BIS-11		65.4 ± 12.1	64.7 ± 11.2	66.1 ± 13.2	0.851
KO scale		31.7 ± 6.1	31.6 ± 6.0	31.9 ± 6.3	0.790
Personality trait					
Avoidant		28.6 ± 20.3	21.4 ± 19.2	35.8 ± 19.3	0.020*
Submissive		27.3 ± 22.1	19.0 ± 18.4	35.7 ± 22.8	0.016*
Asocial		26.8 ± 20.6	23.0 ± 17.6	30.7 ± 23.1	0.443
Conforming		21.3 ± 18.2	17.5 ± 14.5	25.2 ± 21.0	0.279
Negativity		17.3 ± 19.2	11.4 ± 16.3	23.3 ± 20.4	0.047*
Problem indicator		15 ± 20.4	8.9 ± 19.0	21.1 ± 20.4	0.020*
Gregarious		13.8 ± 14.9	13.0 ± 15.3	14.6 ± 14.9	0.696
Previous self-injury history					0.048^*^
Yes		7 (19.4%)	1 (2.8%)	6 (16.7%)	
No		27 (75.0%)	15 (41.7%)	12 (33.3%)	
Do not know		2 (5.6%)	2 (5.6%)	0 (0%)	
Previous suicidal attempt history					> 0.99
Yes		5 (13.9%)	3 (8.3%)	2 (5.6%)	
No		31 (86.1%)	15 (41.7%)	16 (44.4%)	
Recommendation by a close acquaintance for psychiatric counseling or treatment		17 (47.2%)	7 (19.4%)	10 (27.8%)	0.318
Self-request for psychiatric counseling or treatment		12 (33.3%)	6 (16.7%)	6 (16.7%)	> 0.99
Psychiatric intervention history					
Yes 17 (47.2%)	Psychiatric treatment	13 (36.1%)	7 (19.4%)	6 (16.7%)	> 0.99
	Psychological counseling	14 (38.9%)	6 (16.7%)	8 (22.2%)	0.733
	Both	12 (33.3%)	6 (16.7%)	6 (16.7%)	> 0.99
No		19 (52.8%)	9 (25%)	10 (27.8%)	> 0.99
Admission history in psychiatry department					> 0.99
Yes		3 (8.3%)	1 (2.8%)	2 (5.6%)	
No		33 (91.7%)	17 (47.2%)	16 (44.4%)	
Medication treatment before death					0.876
Yes 7 (19.4%)	Regularly	3 (8.3%)	1 (2.8%)	2 (5.6%)	
	intermittent	4 (11.1%)	2 (5.6%)	2 (5.6%)	
No 29 (80.6%)	stop	3 (8.3%)	1 (2.8%)	2 (5.6%)	
	No history	26 (72.2%)	14 (38.9%)	12 (33.3%)	

M: mean; SD: standard deviation; N: number; K-BDI-II: Korean version of Beck Depression Inventory-II; K-ARS-IV: Korean Attention Deficit/Hyperactivity Disorder (ADHD) rating scale-IV; K-BIS-11: Korean version of the Barratt Impulsiveness Scale-11; KO scale: Internet Addiction Proneness Scale for Youth: Observer Rating Scale.

### Suicide warning signs before death

Before death, 80.6% of all adolescents exhibited one or more warning signs of suicide (Table 4). Verbal, behavioral, and emotional signs were observed in 86.2%, 72.4%, and 55.2% of the adolescents, respectively. Approximately half (52.8%) left a suicide note.

**Table 4:** Warning signs before death in adolescents who died by suicide (*N* = 36)

Variables	Total (36, 100%)	Male (18, 50%)	Female (18, 50%)	p value
	M ± SD / N (%)			
Suicide warning sign				0.402
Yes	29 (80.6%)	16 (44.4%)	13 (36.1%)	
No	0 (0%)	0 (0%)	0 (0%)	
Do not know	7 (19.4%)	2 (5.6%)	5 (13.9%)	
Type of suicide warning signs (multiple selection)				
Verbal signs	25 (69.4%)	14 (38.9%)	11 (30.6%)	0.471
Behavioral signs	21 (58.3%)	9 (25%)	12 (33.3%)	0.5
Emotional signs	16 (44.4%)	7 (19.4%)	9 (25%)	0.738
Suicide note				1.0
Yes	19 (52.8%)	10 (27.8%)	9 (25%)	
No	17 (47.2%)	8 (22.2%)	9 (25%)	
Where to leave suicide note (multiple selection)				
Letter	11 (30.6%)	5 (13.9%)	6 (16.7%)	1.0
Memo	2 (5.6%)	1 (2.8%)	1 (2.8%)	1.0
Social network service	4 (11.1%)	3 (8.3%)	1 (2.8%)	0.603
Others (diary and cellular phone)	4 (11.1%)	2 (5.6%)	2 (5.6%)	1.0

M: mean; SD: standard deviation; N: number.

### Word clouds of risk and protective factors

**Fig. 1:**
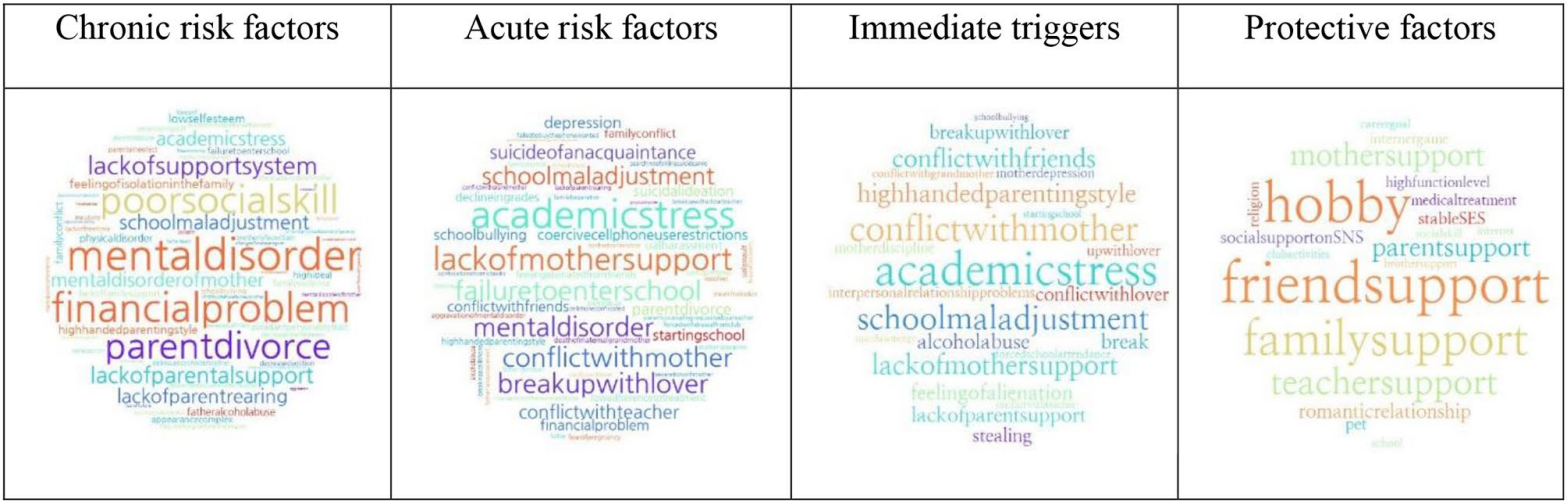
Word clouds of risk and protective factors before death in the adolescents

In Fig. 1, the chronic and acute risk factors, immediate triggers, and protective factors are visually represented as word clouds.

### Information related to school life

Half of the parents (50%) reported that their children were happy with school life (Table 5). Many students participated in school club activities (44.4%) and had close teachers who could be consulted when needed (41.7%). Parents reported that many adolescents experienced academic and peer relationship stresses (69.4% and 58.3%, respectively).

**Table 5:** Information related to the school life of adolescents (*N* = 36)

Variables	Total (36, 100%)	Male (18, 50%)	Female (18, 50%)	p value
	M ± SD / N (%)			
Satisfaction with school life				1.0
Yes	18 (50%)	9 (25%)	9 (25%)	
No	15 (41.7%)	7 (19.4%)	8 (22.2%)	
Unknown	3 (8.3%)	2 (5.6%)	1 (2.8%)	
Participation in a school club				0.715
Yes	16 (44.4%)	7 (19.4%)	9 (25%)	
No	14 (38.9%)	8 (22.2%)	6 (16.7%)	
Unknown	6 (16.7%)	3 (8.3%)	3 (8.3%)	
Academic performance				0.184
High	8 (22.2%)	2 (5.6%)	6 (16.7%)	
Middle	17 (47.2%)	11 (30.6%)	6 (16.7%)	
Low	11 (30.6%)	5 (13.9%)	6 (16.7%)	
An abrupt decline in academic performance before death.				0.428
Yes	3 (8.3%)	2 (5.6%)	1 (2.8%)	
No	24 (66.7%)	10 (27.8%)	14 (38.9%)	
Unknown	9 (25%)	6 (16.7%)	3 (8.3%)	
Academic stress				0.448
Yes	25 (69.4%)	12 (33.3%)	13 (36.1%)	
No	9 (25%)	6 (16.7%)	3 (8.3%)	
Unknown	2 (5.6%)	0	2 (5.6%)	
Stress related with peers				1.0
Yes	21 (58.3%)	10 (27.8%)	11 (30.6%)	
No	11 (30.6%)	6 (16.7%)	5 (13.9%)	
Unknown	4 (11.1%)	2 (5.6%)	2 (5.6%)	
Stress-related with teachers				0.726
Yes	12 (33.3%)	7 (19.4%)	5 (13.9%)	
No	20 (55.6%)	10 (27.8%)	10 (27.8%)	
Unknown	4 (11.1%)	1 (2.8%)	3 (8.3%)	
Presence of an intimate teacher				0.715
Yes	15 (41.7%)	8 (22.2%)	7 (19.4%)	
No	14 (38.9%)	6 (16.7%)	8 (22.2%)	
Unknown	7 (19.4%)	4 (11.1%)	3 (8.3%)	

M: mean; SD: standard deviation; N: number.

### Adverse events during maturation of adolescents

**Fig. 2:**
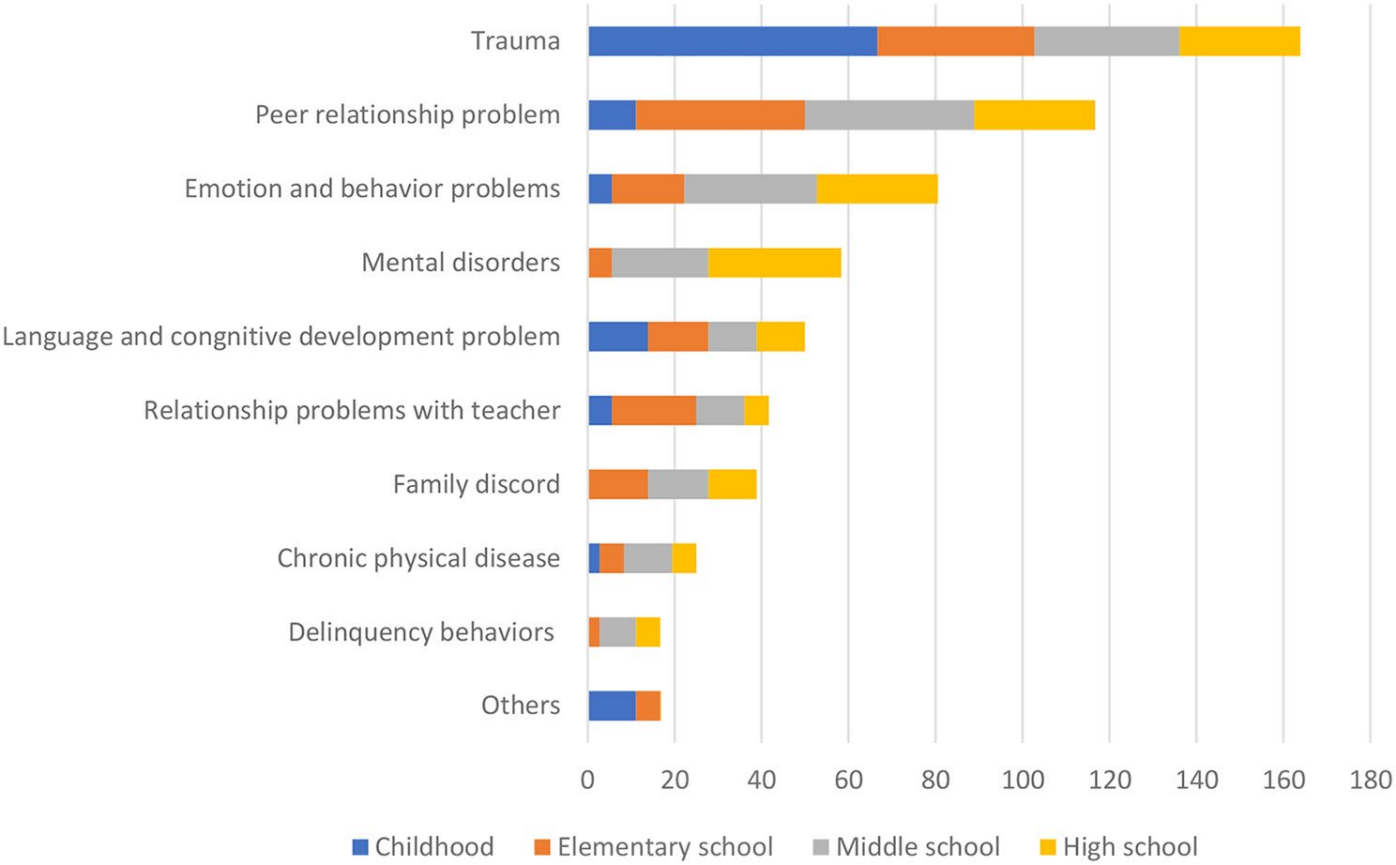
Adverse events based on developmental stage (*N* = 36, %)

Parents mentioned adverse events that may have affected the growth and development of the deceased (Fig. 2). All adolescents experienced one or more adverse events (1.6 ± 0.8); the most common adverse event was trauma. In this study, trauma included trauma related to family (such as parental divorce or separation, family disease or death, family financial problems, and domestic violence) and trauma not related to family (such as natural disasters, illness or death of an acquaintance, physical, verbal, or sexual abuse, and severe crime). Family discord means not a form of trauma, but rather a conflict within a family.

### Information related to the mental health of the family

Many parents (50%) had a psychiatric diagnosis before their offspring died (Table 6). In three generations (including parents and grandparents), 25% had a familial history of suicide. A history of suicide attempt was reported by 2.8% of fathers and 13.9% of mothers.

**Table 6:** Information related to the mental health of the family reported by parents (*N* = 36)

Variables		Total (36, 100%)	Male (18, 50%)	Female (18, 50%)	p value
		M ± SD / N (%)			
Psychiatric Diagnosis of parents		18 (50%)			0.789
Depressive disorder		6 (16.7%)	2 (5.6%)	4 (11.1%)	
Addictive disorder		4 (11.1%)	2 (5.6%)	2 (5.6%)	
Bipolar disorder		3 (5.6%)	1 (2.8%)	2 (5.6%)	
Sleep-wake disorder		2 (2.8%)	1 (2.8%)	1 (2.8%)	
Panic disorder		1 (2.8%)	1 (2.8%)	0 (0%)	
Personality disorder		1 (2.8%)	0 (0%)	1 (2.8%)	
Post-traumatic stress disorder		1 (2.8%)	0 (0%)	1 (2.8%)	
Alcohol abuse history		5 (13.9%)			1.0
Biological father		5 (13.9%)	3 (8.3%)	2 (5.6%)	
Biological mother		0 (0%)	0 (0%)	0 (0%)	
Suicidal attempt history		6 (16.7%)			1.0
Biological father		1 (2.8%)	1 (2.8%)	0 (0%)	
Biological mother		5 (13.9%)	2 (5.6%)	3 (8.3%)	
Suicide death history of three generation					0.264
Yes		9 (25%)	3 (8.3%)	6 (16.7%)	
No		26 (72.2%)	14 (38.9%)	12 (33.3%)	
Unknown		1 (2.8%)	1 (2.8%)	0 (0%)	
Psychiatric intervention history of parents					0.141
Yes 12 (30.6%)	psychiatric treatment	9 (25%)	4 (11.1%)	5 (13.9%)	
	psychological counselling	3 (8.3%)	0 (0%)	3 (8.3%)	
No		23 (63.9%)	14 (38.9%)	9 (25%)	
Decline to answer		1 (2.8%)	0 (0%)	1 (2.8%)	

M: mean; SD: standard deviation; N: number.

## Discussion

This is the first study to analyze psychological autopsy data of 36 adolescents who died by suicide in Korea. It investigated the risk and protective factors related to suicide to understand the adolescent mental health and environment before death and analyzed sex differences. The important findings were, first, most adolescents (97.2%) had at least one major psychiatric disorder, and depressive disorder was the most prevalent. Half of the adolescents (55.6%) had comorbidities of which depressive disorders was the most common. Second, most adolescents (80.6%) expressed suicide warning signs to their family members. Third, 19.4% of the adolescents injured themselves and 13.9% had attempted suicide a year before death. Fourth, Chronic risk factors were mainly related to adolescents’ mental illness and problems related to family, such as parental divorce or financial problems. Immediate triggers included academic stress, conflict with their mother, and school maladjustment. Hobbies and supportive friends, family, and teachers are essential to protect the mental health of adolescents. Fifth, 17% of the adolescents had a biological parent who had attempted suicide, and 25% had a family history of suicide in the three generations. However, the adolescents did not exhibit impulsive behavior or express psychological difficulties; therefore, people around them, including their parents, could not recognize the risk of suicide.

### Suicide method and place

This study found an equal gender distribution among adolescents, contrary to the general trend where male adolescents usually exhibit higher suicide rates influenced by method variations [53, 54]. Men use more lethal methods, such as hanging, jumping, using firearms and gas, while women use less destructive methods, such as drug overdose or cutting their wrists [6, 9, 10, 22, 55–57]. We found no significant gender difference in suicide methods, and jumping from a height was the most common method. As a resulta, the gender-ratio gap in South Korea could be none or lower than that in Western countries [29, 56]. However, the suicide rate among women in rural areas of China and India is increasing due to women’s low social status resulting from traditional hegemonic paternalistic values [9, 58, 59]. In South Korea, gender inequality has improved, but glass ceilings remain; therefore, similar reasons cannot be ruled out completely [60]. Additionally, the suicide rate among female adolescents may have increased because of the Werther effect caused by recent Korean female celebrity suicides [61]. To determine the cause of the increased suicide rate among Korean female adolescents, it is necessary to monitor future trends.

We found that the death of adolescents was caused by the most jumping from height, followed by hanging or strangulation, and poisoning by gas or vapor. The choice of suicide method is influenced by situational accessibility, availability, lethality, and socio-cultural acceptability [62, 63]. Suicide methods vary globally. Pesticide ingestion is common in rural China, while jumping in front of moving objects is widespread in countries with advanced railway systems [58, 64]. Firearm suicides are prevalent in rural Australia and the United States [62, 65]. In urbanized countries like Korea, jumping from a height is a common method of suicide, which is also the case in Singapore and Hong Kong [31, 66]. Overall, hanging, pesticide poisoning, and firearms are standard methods across age groups worldwide, with hanging predominating among Korean adults [1–3]. Notably, falls from a height are prominent among Korean adolescents. In this study, most suicides took place at home. Similar findings were observed in other countries [28, 67]. People may perceive that it is easier to die by suicide at home, as it is a private space [28].

### Psychiatric characteristics

Most of the adolescents were diagnosed with major mental disorders (97.2%) according to the DSM-5 [68], and affective disorders (80.6%). Depressive disorders were the most common psychiatric disorder (70%). Generally, female adolescents who died by suicide are more prone to internalizing issues like depressive disorder, while male adolescents are more likely to have externalizing problems such as conduct disorder [69–71]. However, we found no gender differences in mental disorders. Teachers’ report for adolescents who died by suicide, which had a larger sample size than that of this study, showed the same results [71, 72]. Parents rated that the adolescents had severe depression (K-BDI-II = 24.2, SD, 13.6). Therefore, increasing access to specialized treatment for adolescents experiencing psychological difficulties can help prevent suicide.

In the Western countries [73, 74], adolescents who died by suicide had a high rate of alcohol or substance abuse. Alcohol consumption is a significant contributor to acute suicide attempts [29]. Although there was no primary diagnosis in our study, alcohol abuse was observed in only one adolescent (2.8%). Asian countries, including Singapore and China, have lower rates of alcohol and substance abuse than Western countries [29, 75].

### Treatment history

Among the adolescents in our study, half (47.2%) had a history of psychiatric intervention (psychological counseling or psychiatric treatment) at least once. Studies in the United Kingdom [73], Singapore [29], and New Zealand [24] found that 14–39% of adolescents contacted a mental health specialist the year prior to their death. Although the number of Korean adolescents who contacted mental health specialists before death was higher than that in other countries, it does not mean that all adolescents received the appropriate treatment, and just contact with specialists did not prevent suicide. In our study, only three of 10 adolescents who received medical treatment continued to take their medication regularly. Among the study participants, 97.2% of adolescents had significant mental disorders. However, providing appropriate psychiatric services is challenging because adolescents are typically reluctant to seek help [76]. Supportive relationships with teachers, parents, friends, and healthcare providers facilitate access to mental health treatment [76]. Therefore, it is essential to teach all adolescents, teachers, parents, and peers to recognize suicide warning signs, and how to receive appropriate treatment. The Korean gatekeeper training program, Suicide CARE, could be an appropriate learning tool for this purpose [37].

### Impulsivity and excessive internet use

Impulsivity is highly correlated with suicidal behavior regardless of the presence or absence of psychiatric disorders [77–80]. We found that the adolescents did not exhibit clinically high impulsiveness, which contradicts previous research findings [77–80]. This might be due to differences in study design. Most previous studies have focused on suicide attempters rather than suicide completers and the participants were adults instead of adolescents [77–80]. Meanwhile, multivariate analysis found that impulsiveness do not directly predict suicide in adolescents who died by suicide [26]. Therefore, impulsiveness may be a symptom of mood disorders or other mental disorders, rather than a significant risk factor for suicide [26, 81].

The adolescents had a high risk of Internet addiction before death. The relationship between excessive Internet use and suicide risk has been reported in Korea and other countries [82–85]. Excessive Internet use may serve as a coping strategy or be influenced by deindividuation and contagion effects [82–84]. While some studies highlight the potentially positive effects of social networking systems (SNS) for adolescents, creating a safe online environment is crucial to enhancing these benefits [83, 85]. To mitigate the adverse effects of SNS and amplify the positive impact on adolescents at risk of suicide, it is imperative to examine the influence of SNS content on their risk of suicide and develop effective intervention strategies accordingly.

### Personality trait

Avoidance (28.6%) and submissiveness (27.3%) were the two most common personality traits among the adolescents. Particularly, it was significantly higher in female adolescents than in male adolescents. Previous studies have reported that mostly borderline/ neurotic/ impulsive/ hostile/ antisocial personality traits corresponding to Cluster B personality traits are related to suicide risk [80, 86–90]. Most studies [11, 80, 86–89] have focused on identifying risk factors for suicide attempters, not suicide completers. Some studies targeting people who died by suicide have shown inconsistent results regarding personality traits [11, 91–93]. One study reported that suicide was related to comorbid conditions rather than personality disorders, while other studies indicated that it was related to borderline or other personality disorders [91–93]. However, some studies also mention that avoidant traits are related to suicide completers [87, 90]. Notably, one study [94] were diagnosed with a personality disorder, with dependent personality disorder being the most common. Further research is needed to confirm the prevalence of avoidant or submissive personality traits in Korean adolescents who die by suicide and explore the significance of these traits, particularly in females.

### Suicide warning sign

Most adolescents who died by suicide had never attempted suicide, and only 13.9% had one or more previous suicide attempts. This percentage is similar to or slightly higher in other countries than that in Korea [24, 28, 29, 75, 95, 96]: 22.9% in Singapore [29], 26.6% in rural China [75], 13.1% in New Zealand [24], 20% in Canada [95], 14.3% in Mexico [28], and 30% in Finland [96]. As most adolescents who die by suicide (approximately 80%) use lethal methods during their first suicide attempt, the rate of previous suicide attempts (13.1–30%) is lower than that of adolescents who make repeated suicide attempts (37.9–53.5%) [97–103]. The rate of death by suicide after a first attempt is higher in adolescents than in adults [28, 38, 104].

Individuals who died by suicide expressed their intentions before dying by suicide. About half of the deceased (52.8%) left a suicide note before death in a diary, memo, online post, or social network service. Studies in Canada [105] and New Zealand [24] also showed that 30–40% of deaths left a suicide note. We identified verbal, behavioral, and emotional warning signs of suicide, and parents recognized suicide warning signals in 80.6% of cases during psychological autopsies. In teachers’ reports on adolescents who died by suicide, warning signs were identified in approximately 40% of the students [104, 106]. A psychological autopsy study on adolescents in the Netherlands also found that 65% of them expressed their suicidal ideation, intent, or behaviors to another person before death [107]. However, these warning signs are often similar to depressive symptoms or non-specific symptoms commonly observed in students without suicidal thoughts [106]. Therefore, it is difficult to identify suicide risk signs according to the progression of depressive symptoms.

Additionally, suicide warning signs are often not disclosed to families or teachers, which may be related to the personality traits of adolescents. Individuals with avoidant or submissive personality traits tend to adjust to or avoid their environment rather than expressing their thoughts or opinions which can make it difficult for the people around them to recognize their problems or difficulties. Moreover, owing to the Confucian culture of Korean society, it is polite and better to conform to societal norms by following the opinions of teachers and adults rather than expressing oneself [108]. As a result, adolescents’ behaviors based on their personality traits are often misunderstood as mature attitudes.

### School life

In our study, 69.4% of the deceased experienced academic stress and 58.3% experienced peer relationship stress. Previous East Asian studies have shown that academic stress increases suicide risk in adolescents [109, 110]. As most adolescents suffered from depression before death, it was expected that the symptoms would have made academics and school life difficult and stressful, and caused them maladjustment at school, such as tardiness or sleeping in class. However, 50% of the adolescents were doing well in school, 44.4% were involved in a school club, 68.4% achieved average or above-average academic performance, and 41.7% had teachers with whom they felt a close connection. Similar to our findings in previous studies, most adolescents who died by suicide had a strong sense of school belongingness and good relationships with friends and teachers [104, 106, 111]. Parents reported that the adolescents who died by suicide were characterized by avoidance, submissiveness, and asocial traits. Adolescents would try to conceal their emotions and ideas and adapt to their environment, which may make it challenging for teachers to identify any changes in their behavior before they died by suicide. According to teacher reports on adolescents who died by suicide, the identification rate of suicide warning signs by teachers was 40.5%, which was half of the rate (80.6%) reported by parents [106]. Therefore, it is essential to educate suicide CARE into gatekeeper training for teachers, adolescents, and school officials, enabling them to detect early warning signs of suicide and implement therapeutic interventions.

### Family

We found that the family history of suicide deaths was 25% across three generations. In addition, many parents were diagnosed with psychiatric disorders (50%) and had a history of suicide attempt (16.7%). Similarly, studies in other countries targeted two or three generations and reported the family history of suicide deaths from 8 to 46.4% [24, 28, 55, 96, 112–114]. Many researchers have consistently reported that suicide attempts have high familial transmission rates, similar to psychopathology [115–118]. Suicidal behavior and psychopathology of the offspring are influenced by the genetic factors of the parents, as well as environmental risk factors, such as marital and parent–child conflicts due to a lack of problem-solving skills and poor communication [28, 115, 118].

In our previous study [119], many adolescents (74.3%) experienced family related trauma, with parental divorce or parent-child separation being the most common (54.3%). In this study, the parents reported that 40% of the deceased experienced family discord during their developmental stage. Word clouds also showed that many chronic risk factors referred to family-related adverse events. Studies in New Zealand [24] and Mexico [28] support our results that 60–70% of adolescents experienced dysfunctional family. Therefore, to prevent adolescent suicide, it is crucial to enhance resilience and provide psychosocial care for adolescents who have experienced adverse life events. Additionally, family-based support should supplement care aimed at adolescents.

## Limitations

This study has several limitations. First, the sample size was small; therefore, it is difficult to generalize the findings to all adolescents who died by suicide in South Korea. Second, no control group was included. It is essential to have caution when interpreting some of the findings presented in this study. Especially, protective factors were defined as factors recognized by parents that acted as a buffer for adolescents who died by suicide in difficult situations before death. Therefore, it is difficult to interpret it as a factor involved in preventing suicide among adolescents. Third, the K-SADS-PL-K, K-BIS-11, and K-BDI-II are assessment tools to be completed by adolescents, but parents completed them in this study because diagnostic information for adolescents who died by suicide is most often obtained from parents [120]. However, reliance on parental recall may lead to potential overestimation or underestimation of the data. Fourth, as this is a postmortem and retrospective study, limitations may arise due to recall bias. Conducting a psychological autopsy with two or more interviewees, particularly involving peers in the case of adolescents, can offer valuable insights into suicide [121]. Despite potential ethical and economic challenges, involving peers in psychological autopsies can be a practical method for screening and intervening with adolescents affected by a friend’s suicide. This approach should be considered in future studies.

## Conclusions

This is the first study exploring the risk and protective factors using psychological autopsy data of adolescents who died by suicide in Korea. We found that most of the adolescents had mental disorders. They were not highly impulsive and were continuously exposed to adverse family-related events during their developmental years. Adolescent expressed their suicidal intentions using verbal and nonverbal warning signs before dying by suicide. However, it was challenging to identify the risk factors before death because (1) the adolescents had avoidant and submissive personality traits and were not to express their feelings and thoughts to others, and (2) they did not exhibit behavioral problems. To prevent adolescent suicide, parents, teachers, and adolescents should be educated about suicide warning signs, so that they can recognize psychological difficulties and take psychiatric treatment. Additionally, a structured educational program is needed to raise awareness about youth’s mental health status and encourage help-seeking.

### Electronic supplementary material

Below is the link to the electronic supplementary material.

Supplementary Material 1

## Data Availability

Not applicable.

## Abbreviations

K-PAC-A: Korean Psychological Autopsy Checklist for Adolescents
K-SADS-PL-K: Kiddie-Schedule for Affective Disorders and Schizophrenia-Present and Lifetime-Korean Version
DSM-IV: Diagnostic and Statistical Manual of Mental Disorders-IV
DSM-5: Diagnostic and Statistical Manual of Mental Disorders-5
K-BIS-11: Korean Version of the Barratt Impulsiveness Scale-11
K-BDI-II: Korean version of Beck Depression Inventory-II
K-ARS-IV: Korean ADHD rating scale-IV
KO scale: Internet Addiction Proneness Scale for Youth: Observer Rating Scale
