# Supplementary material for: Suicide warning signs that are challenging to recognize: a psychological autopsy study of Korean adolescents
Source: Child Adolesc Psychiatry Ment Health. 2024 Mar 25;18:41. doi: 10.1186/s13034-024-00731-1 (PMC10964623; doi:10.1186/s13034-024-00731-1)
Supplement: Supplementary file 1 — Supplementary Material 1 [file 13034_2024_731_MOESM1_ESM.docx]

Appendix 1. A brief overview of the Korean Psychological Autopsy Checklist for Adolescents

| Parents of adolescents who died by suicide (interviewee) | Part Ⅰ | Part Ⅲ |
| --- | --- | --- |
|  | - Sociodemographic information - Psychological and adaptive status after the adolescent’s death | - Patient Health Questionnaire–9 - Korean version of the Insomnia Severity Index - Alcohol Use Disorders Identification Test–Concise |
| Adolescents who died by suicide | Part Ⅱ | Part Ⅲ |
|  | - Sociodemographic information - Age at the time of death - Method of suicide - Place of suicide - Suicide warning signs - History of suicidal behaviors - Developmental history - Adverse events based on the developmental stage - Personality traits - Relationships with parents, peers, and teachers - Family-related information - School life-related information - Physical and mental health | - Kiddie-Schedule for Affective Disorders and Schizophrenia-Present and Lifetime–Korean Version - Korean version of the Beck Depression Inventory-II - Korean Attention Deficit/Hyperactivity Disorder Rating scale - Internet Addiction Proneness Scale for Youth: Observer Rating Scale - Korean version of the Barratt Impulsiveness Scale-11 |

Appendix 2. In-depth analysis form of adolescent psychological autopsy

| Study number |  | Interview date and time |  |
| --- | --- | --- | --- |

| Key words for adolescents who died by suicide  (less than 5) | A brief description of adolescents who died by suicide |
| --- | --- |

| General information | |
| --- | --- |
| Gender/Age |  |
| Family members and  relationship description |  |
| School life |  |
| Others |  |

| Suicide-related behaviors and information related to death | |
| --- | --- |
| Time of death |  |
| Place and method |  |
| Suicide warning signs and asking for help after suicidal behavior before death |  |
| Previous suicidal ideation or attempt history |  |
| Suicide note |  |
| Others |  |

| Developmental history and adverse events based on developmental stage | |
| --- | --- |
| Personality traits and talents |  |
| Birth |  |
| Toddlerhood |  |
| Childhood |  |
| Adolescence |  |
| Others |  |

| Suicide warning signs according to time flow before death | |
| --- | --- |
| One~two years ago |  |
| One~two months ago |  |
| One week ago |  |
| One day ago |  |
| The day |  |
| Others |  |

| School life and relationships with peers and teachers | |
| --- | --- |
| Academic stress |  |
| Relationships with peers |  |
| Relationships with teachers |  |
| Others |  |

| Romantic and interpersonal relationships | |
| --- | --- |
| Romantic relationships |  |
| Interpersonal relationships |  |
| Others |  |

| Physical and mental health | |
| --- | --- |
| Physical health |  |
| Mental health |  |
| Family history of mental health |  |
| Suicide/self-injurious behavior of friends or acquaintances |  |
| Others |  |

| Diagnosis according to the Kiddie-Schedule for Affective Disorders and Schizophrenia-Present and Lifetime-Korean Version | |
| --- | --- |
| Main diagnosis |  |
| Comorbidities |  |

| Risk and protective factors | |
| --- | --- |
| Chronic risk factors |  |
| Acute risk factors |  |
| Immediate triggers |  |
| Protective factors |  |

| Retrospective Suicide Estimated Pathways |
| --- |
|  |

| Psychodynamic formulation |
| --- |
|  |

Appendix 3. Research team meeting process

DSM-5: Diagnostic and Statistical Manual of Mental Disorders-5th edition

Appendix 4. Suicide Warning Signs

| Category | Contents |
| --- | --- |
| Verbal signs | often talked about suicide, homicide, and death |
|  | reported experiencing physical discomfort |
|  | made self-deprecating remarks |
|  | asked questions about how to commit suicide |
|  | longed for the afterlife |
|  | talked about people who had committed suicide |
|  | documented deaths in letters, retreat records, notebooks, and other formats |
| Behavioral signs | changed in sleep patterns: insomnia or hypersomnia |
|  | changed in appetite: appetite increases excessively or decreases even if not on a diet |
|  | put their house in order |
|  | made a suicide plan |
|  | observed a decline in their performance due to unusual and, sometimes, bizarre behavior |
|  | diminished concentration or indecisiveness |
|  | neglected personal grooming |
|  | self-injurious behavior or substance abuse |
|  | excessive engagement with music, poetry, and movies related to death |
|  | apologized for past mistakes and attempted to mend broken relationships |
|  | gave away cherished possessions to loved ones |
| Emotional signs | changed in emotional state: guilt, shame, loneliness, instability, helplessness, and hopelessness |
|  | lethargy, avoidance of people, and loss of interest |
